# Genetic deficiency of Wnt5a diminishes disease severity in a murine model of rheumatoid arthritis

**DOI:** 10.1186/s13075-017-1375-0

**Published:** 2017-07-19

**Authors:** Susan MacLauchlan, Maria A. Zuriaga, José J. Fuster, Carla M. Cuda, Jennifer Jonason, Fernanda Behzadi, Jennifer Parker Duffen, G. Kenneth Haines, Tamar Aprahamian, Harris Perlman, Kenneth Walsh

**Affiliations:** 1 Molecular Cardiology, Whitaker Cardiovascular Institute, Boston University School of Medicine, 700 Albany Street, W-611, Boston, MA 02118 USA; 2 Division of Rheumatology, Department of Medicine, Northwestern University, Feinberg School of Medicine, 240 E. Huron Street, McGaw M338 Chicago, IL USA; 3 University of Rochester Medical Center, School of Medicine and Dentistry, 601 Elmwood Ave, Box 665, Rochester, NY USA; 4 Renal Section, Boston University School of Medicine, 650 Albany St, Boston, MA USA; 5 Department of Pathology, Icahn School of Medicine at Mount Sinai, New York, NY 10029 USA

**Keywords:** Rheumatoid arthritis, Wnt5a, Inflammation, Osteoclast fusion

## Abstract

**Background:**

Rheumatoid arthritis (RA) is a common autoimmune disease characterized by chronic inflammation of the joints, leading to bone erosion and joint dysfunction. Despite the recent successes of disease-modifying anti-rheumatic drugs (DMARDs), there is still clinical need for understanding the development and molecular etiology of RA. Wnts are developmental morphogens whose roles in adult pathology are poorly characterized. Wnt5a is a member of the non-canonical family of Wnts that modulates a wide range of cell processes, including differentiation, migration, and inflammation. Wnt5a has been implicated as a possible contributor to arthritis and it is upregulated in synovial fibroblasts from RA patients.

**Methods:**

We investigated the role of endogenous Wnt5a in RA. Tamoxifen-inducible, Wnt5a knockout (Wnt5a cKO) mice and littermate controls were monitored for arthritis development and joint pathology using the K/BxN serum transfer-induced arthritis (STIA) model. To explore a role of Wnt5a in osteoclast fusion, bone marrow-derived monocytes (BMDMs) were differentiated in vitro.

**Results:**

Wnt5a cKO mice were resistant to arthritis development compared to control littermates as assessed by ankle thickness and histologic measurements. Some parameters of inflammation were reduced in the Wnt5a cKO mice, including the extent of polymononuclear cell infiltration and extra-articular inflammation. Wnt5a cKO mice also exhibited less cartilage destruction and a reduction in osteoclast activity with concomitant reduction in *tartrate-resistant acid phosph﻿atase* (*TRAP*), *cathepsin K* (*CTSK*), *macrophage colony-stimulating factor* (*MCSF*), *matrix metalloproteinase* (*MMP*)*2* and *MMP9* in the arthritic joints. Treatment of BMDMs with Wnt5a enhanced osteoclast fusion and increased the expression of *dendrocyte-expressed seven transmembrane protein* (*DCSTAMP*) and *MMP9*, that are necessary for osteoclast formation and activity.

**Conclusions:**

These data suggest that Wnt5a modulates the development of arthritis by promoting inflammation and osteoclast fusion, and provide the first mouse genetic evidence of a role for endogenous Wnt5a in autoimmune disease.

**Electronic supplementary material:**

The online version of this article (doi:10.1186/s13075-017-1375-0) contains supplementary material, which is available to authorized users.

## Background

Rheumatoid arthritis (RA) is a common chronic autoimmune disease that leads to significant morbidity and mortality. The prevalence of this disease is between 0.5 and 1% in the USA, with greater risk of incidence among women [1]. The molecular cues that trigger RA are complex and likely require a combination of genetic and environmental factors. Ultimately, the loss of self-tolerance to self-proteins, typically citrullinated-proteins, results in disease progression through inflammation, synovial hyperplasia, and osteoclast activation [2]. These processes lead to the recognizable hallmarks of the disease, including joint swelling, pain, and dysfunction. Although joint involvement is the major clinical manifestation of the autoimmune process, RA patients suffer from a number of co-morbidities including increased risk of cardiovascular disease even when accounting for the increased inflammatory state [3, 4]. Significant strides have been made in the treatment of RA. Standard therapy for RA includes methotrexate and one of several disease-modifying anti-rheumatic drugs (DMARDs) that target the key inflammatory molecules that promote RA, including TNFα (infliximab, etanercept) and IL6 (tocilizumab). These treatments allow many patients to achieve disease-free status, but their use is associated with significant side effects and these biologic agents do not confer sustained remission after drug cessation [1, 5]. Thus, there remains a clinical need for improved understanding of novel targets for drugs in the process of RA development.

The Wnt family proteins are developmental regulators that are becoming appreciated for their diverse roles in adult physiology. Wnt5a is the archetypal non-canonical Wnt, the subset of Wnts whose functions are independent of beta-catenin [6]. In post-natal tissue, the functions of Wnt5a are diverse and include modulation of cell fate decisions and inflammation. Previous work has established Wnt5a as a pro-inflammatory molecule in a number of settings including vascular dysfunction [7, 8], peripheral arterial disease [9], and cardiometabolic disease [10–12]. Wnt5a has also been demonstrated to promote expression of various cytokines that may contribute to RA progression [13–16]. However, studies on the role of Wnt5a in immune regulation can be confounded by the likely presence of TLR4 agonists in commercial Wnt5a protein preparations [17]. Thus, analyses of Wnt5a function using mouse genetic models are warranted as they are not subject to this potential confounder.

Wnt5a is increased in the synovium in patients with RA and osteoarthritis (OA) [18], and inhibition of Wnt5a is reported to block the activation of cultured synovial fibroblasts from RA patients [19]. In addition, the delivery of a soluble form of receptor-tyrosine orphan receptor 2 (ROR2), a candidate decoy receptor for Wnt5a, leads to reduced radiographic severity in mice undergoing collagen-induced arthritis (CIA) [20]. These data underscore the potential clinical value of Wnt5a in the pathogenesis of RA. However, there is as yet no direct genetic evidence to support that endogenous Wnt5a plays a contributory role in the development of RA.

In the present study, we used inducible Wnt5a knockout (Wnt5a cKO) and littermate control mice in the K/BxN serum transfer-induced arthritis (STIA) model. Wnt5a cKO mice were protected from development of RA, both in terms of ankle thickness and other markers of disease severity. Histologic examination demonstrated reduced inflammation in the Wnt5a cKO, including reduced PMN infiltrate into the inflamed joints. Less osteoclast activity was also observed in the arthritic Wnt5a cKO joints compared to the controls. Mechanistically, this may result from decreases in osteoclast fusion as Wnt5a promoted osteoclast fusion in an in vitro culture of bone marrow-derived macrophage (BMDM) differentiation. Wnt5a increased the expression of critical molecules for osteoclast differentiation and fusion, specifically *dendrocyte-expressed seven transmembrane protein* (*DCSTAMP*) and *matrix metalloproteinase 9* (*MMP9*). These data suggest Wnt5a signaling as a modulator of inflammation and osteoclast fusion could represent a component of disease activity in RA patients.

## Methods

### Animals

All animal protocols were approved by the Institutional Animal Care and Use Committee (IACUC), Boston University School of Medicine (BUSM). Wnt5a whole body inducible cKO mice (Wnt5a cKO) were generated as previously described [10] by selective breeding of the *Wnt5a* floxed mice [21] with the *UBC* cre promoter mice (Jackson Labs, Bar Harbor, ME, USA, stock number 007001). Female littermate *Wnt5a* floxed cre-negative mice (control) and cre-positive mice (Wnt5a cKO) were used for this study. *Wnt5a* recombination was induced in the mice at 6 weeks of age by five daily intraperitoneal injections of tamoxifen delivered in corn oil (80ug tamoxifen per g body weight each day). Both control and Wnt5a cKO mice were treated with tamoxifen. Baseline mice (at least four per genotype) were euthanized following 2 weeks of tamoxifen wash out. Tissue was collected and processed as described in the following section. Mice were used in the arthritis model following 2 weeks of tamoxifen wash out (*n* = 13 per genotype for RA studies). Female C57BL6J (Jackson Laboratories) mice 10 − 12 weeks of age were used for the in vitro fusion assays (*n* = 3).

### K/BxN serum transfer-induced arthritis (STIA) model

Rheumatoid arthritis development was induced in control and Wnt5a cKO mice using the K/BxN serum transfer model of arthritis as previously described [22]. Briefly, serum from K/BxN mice (the offspring of mice expressing the KRN T cell receptor transgene and the major histocompatibility complex (MHC) class II molecule A(g7), which develop spontaneous arthritis) was isolated at 8 weeks of age. Passive transfer of arthritis was induced in control and Wnt5a cKO mice by intraperitoneal injection of 150 μl of K/BxN serum at day 0 (d0) and d2. Severity of arthritis was monitored by two measures prior to serum injection and every other day for 28 days. The clinical score of each joint was determined using a well-established scoring metric [22–24]. The reported clinical score is the summation of the scores for all four appendages. Ankle thickness was measured at the thickest point of the ankle using digital Vernier calipers (Fine Science Tools). Significance for the clinical score and ankle thickness measurements was determined using the statistical package in Prism GraphPad using two-way analysis of variance (ANOVA). Significance was determined using the Sidak post-hoc test. Tissues were collected at d7 (*n* = 6 per genotype) or d28 (n = 7 per genotype) following induction of arthritis. Serum was collected by cardiac puncture after euthanasia by carbon dioxide inhalation. Paw joints were snap frozen for mRNA isolation and ankle joints were fixed in 10% formalin and decalcified in 0.375 M EDTA.

Paw joints were pooled for each individual animal prior to mRNA extraction. The joints were cleaned of skin, homogenized with Qialyzer (Qiagen) and mRNA was isolated with the Qiagen RNeasy Fibrous tissue kit according to the manufacturer’s directions. cDNA was transcribed from 1 μg of RNA using High Capacity cDNA Kit (Applied Biosystems) and quantitative PCR (qPCR) was performed using SYBR-based detection (primer sequences are shown in Table 1). Fold changes were calculated after normalization of the average *hypoxanthine guanine phosphoribosyl transferase* (*HPRT*) and beta-actin relative to baseline mice. *P* values ≤0.05 from the *t* test calculated using GraphPad Prism were considered significant.

**Table 1:** Sequences for SYBR qPCR

Gene ID	Forward primer	Reverse primer
*CAR2*	TCC CAC CAC TGG GGA TAC AG	CTC TTG GAC GCA GCT TTA TCA TA
*CD68*	TGT CTG ATC TTG CTA GGA CCG	GAG AGT AAC GGC CTT TTT GTG A
*CLCN7*	CGC CAG TGT CAT TCT GCA CT	GCT TCT CGT TGT GTG GAA TCT
*CTSK*	GAA GAA GAC TGA CCA GAA GCA G	TCC AGG TTA TGG GCA GAG ATT
*DCSTAMP*	GGG GAC TTA TGT GTT TCC ACG	ACA AGG CAA CAG ACT CCC AAA T
*F4/80*	CTT TGG CTA TGG GCT TCC AGT C	GCA AGG AGG ACA GAG TTT ATC GTG
*GAPDH*	TGA CCA CCA TGG AGA AGG C	GCT AAG CAG TTG GTG GTG CA
*HPRT*	TCA GTC AAC GGG GGA CAT AAA	GGG GCT GTA CTG CTT AAC CAG
*IL1β*	TGA CAG TGA TGA GAA TGA CCT GTT C	TTG GAA GCA GCC CTT CAT CT
*IL6*	GCT ACC AAA CTG GAT ATA ATC AGG A	CCA GGT AGC TAT GGT ACT CCA GAA
*MCP1*	GCC TAC TCA TTG GGA TCA TCT TG	CAG CCA GAT GCA GTT AAC GC
*MCSF*	GGC TTG GCT TGG GAT GAT TCT	GAG GGT CTG GCA GGT ACT C
*MMP2*	CAG GGT GGT GGT CAT AGC TAC TT	GAG ACT TTG GTT CTC CAG CTT CA
*MMP9*	CAA GTG GGA CCA TCA TAA CAT CA	CTC GCG GCA AGT CTT CAG A
*MMP14*	AGT GAC AGG CAA GGC TGA TTT	AGG GGT GTA ATT CTG AAT GCA G
*NFATc1*	CAG GGC GAG TTC GAC TTC G	TGA CAC TAG GGG ACA CAT AAC TG
*OPG*	ACC CAG AAA CTG GTC ATC AGC	CTG CAA TAC ACA CAC TCA TCA CT
*OSCAR*	CCT AGC CTC ATA CCC CCA G	CGT TGA TCC CAG GAG TCA CAA
*RANKL*	CAG CAT CGC TCT GTT CCT GTA	CTG CGT TTT CAT GGA GTC TCA
*RANK*	GGA CGG TGT TGC AGC AGA T	GCA GTC TGA GTT CCA GTG GTA
*RCAN2*	CCA CTC TGG TCG CCT GTG T	CGG AAC AGT CCC TCG AAT TTT TCC TTA
*TBP*	CTT CCT GCC ACA ATG TCA CAG	CCT TTC TCA TGC TTG CTT CTC TG
*TNFα*	CGG AGT CCG GGC AGG	GCT GGG TAG AGA ATG GAT GAA
*TRAP*	CAC TCC CAC CCT GAG ATT TGT	CAT CGT CTG CAC GGT TCT G
*Wnt5a Ex2*	CAA ATA GGC AGC CGA GAG AC	CTC TAG CGT CCA CGA ACT CC
*18S*	AAT CAA GAA CGA AAG TCG GAG G	GCG GGT CAT GGG AAT AAC G
*36B4*	GCT CCA AGC AGA TGC AGC A	CCG GAT GTG AGG CAG CAG

### Histopathologic assessment

Paraffin-embedded tissue sections were stained with hematoxylin and eosin (H&E). Disease severity was analyzed by a pathologist (KGH) blinded to the genotypes as previously described [25] (n = 6 per genotype). Clinical score, cartilage destruction, bone erosion, inflammation, polymononuclear (PMN) infiltration, extra-articular inflammation, lymphocyte infiltration, pannus formation and synovial lining average were evaluated as previously described [23, 25–28]. Tartrate acid resistant phosphatase (TRAP) staining was performed as specified by the manufacturer (Sigma Kit) (*n* = 6 per genotype at d7 and at least 4 per genotype for baseline analysis). Stained sections were visualized on a Keyence BZ-X700 and analyzed with the on-board image processing software to identify total TRAP+ area in at least four fields per mouse in both ankle joints.

### L cell conditioned media

L control cells (ATCC, CRL-2648) and L Wnt5a cells (ATCC, CRL-2814) were used to generate controls and Wnt5a conditioned media as previously described [29]. L cells were maintained in DMEM with 10% FBS and pen/strep/Lglut. Conditioned medium for the fusion assays were generated in complete alpha-MEM with 10% FBS and pen/strep/Lglut. This conditioned medium was diluted 1:4 with fresh complete alpha-MEM medium for the fusion assays.

### In vitro analysis of fusion

BMDM were isolated from female C57BL6J mice (Jackson Laboratories) and cultured in alpha-MEM with 10% FBS and pen/strep/Lglut. Following differential plating to purify monocyte cells in the presence of macrophage colony-stimulating factor (MCSF) (100 ng/mL), 10^5 cells per well were plated in 24 well plates﻿ in the presence of diluted L cell control or diluted Lcell Wnt5a-treated medium. BMDM were induced to fuse with the stimulation cocktail (MCSF (50 ng/mL) and RANKL (50 ng/mL)), which was replenished at d2 and d4. RNA was isolated at d2 and d5 using the miRNeasy Micro Kit (Qiagen) and 1 μg of RNA was transcribed using the Quantitect kit (Qiagen). SYBR chemistry was used to detect transcripts and these were analyzed on a ViiA7 using the primers listed in Table 1. Significance was determined in GraphPad Prism using two-way ANOVA with the Tukey post-hoc test, considering *p* values ≤0.05 as significant. To enumerate osteoclasts, d5 cultures were stained for TRAP (Sigma) and co-stained with 4',6-diamidino-2-phenylindole (DAPI) (Thermo Fisher Scientific). Cultures were visualized using a Keyence microscope. Osteoclasts were identified as TRAP+ multinucleated cells (3 nuclei or more). Total nuclei, total TRAP+ cells, total osteoclasts and total nuclei in osteoclasts were enumerated in at least six random high powered fields per condition per experiment. The osteoclast frequency per 1000 cells was calculated as the ratio of total osteoclasts per high-powered field (HPF) divided by total nuclei in the same HPF. The percentage of cells in osteoclasts is the total nuclei in osteoclasts per HPF divided by the total nuclei in the same image. Statistical significance for enumeration tests was performed using Prism Software and the unpaired *t* test and considering significance at *p* ≤ 0.05. All in vitro studies were performed three times. Enumeration studies were performed in duplicate three times.

## Results

### Wnt5a-deficiency attenuates the development of RA-like disease

**Fig. 1:**
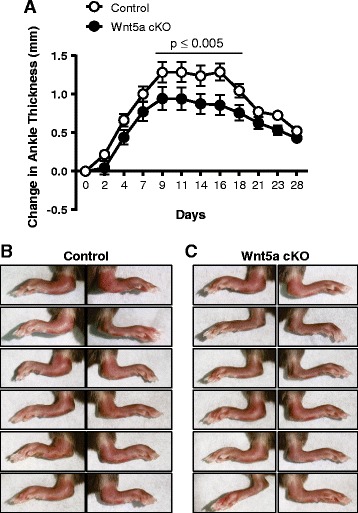
Whole body inducible Wnt5a knockout (*Wnt5a cKO*) mice display attenuated rheumatoid arthritis development in the serum transfer model. **a** The change in ankle thickness was monitored in Wnt5a cKO and littermate control mice following a period of 28 days after induction of arthritis by serum transfer. Images of the ankles at day 7 indicate that there is more inflammation and arthritis in the limbs of control mice (**b**) compared to the Wnt5a cKO mice (**c**)

**Fig. 2:**
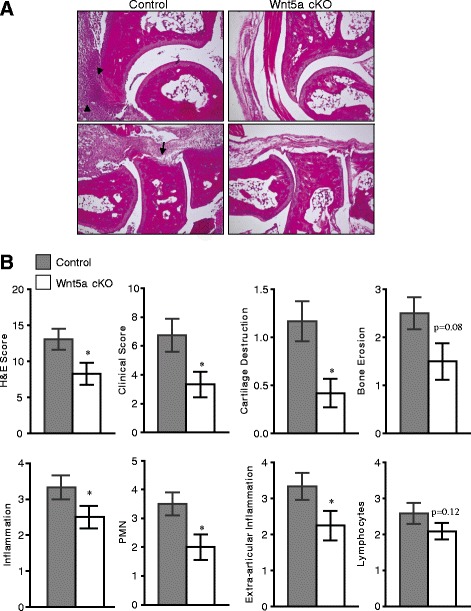
Whole body inducible Wnt5a knockout (*Wnt5a cKO*) mice develop less severe rheumatoid arthritis (RA) based upon histological analyses. **a** H&E-stained sections from the ankles in control and Wnt5a cKO mice at day 7 after induction of arthritis demonstrate hallmark features of the disease at the tarsotibial joint (*top panels*) and the tarsonavicular joint (*bottom panels*), including expanded pannus formation (*arrowheads*) and destruction pits (*arrow*). **b** Qualitative metrics of the damage induced by RA in control and Wnt5a cKO mice demonstrates reduced overall disease severity (H&E score), clinical score, cartilage destruction, inflammation, polymononuclear (*PMN*) cell infiltration and extra-articular lesions in the joints of Wnt5a cKO mice. *P ≤ 0.05

Due to the association of Wnt5a with clinical arthritis, we sought to determine its role in the murine STIA model of RA. In apparent contrast to clinical arthritis [18], *Wnt5a* transcript levels were reduced at d7 after RA induction (Additional file 1: Figure S1). However, active RA induced the levels of the Wnt5a co-receptor *ROR2* mRNA at this time point (Additional file 1: Figure S1). To further evaluate the role of Wnt5a in the development of RA, a murine model of Wnt5a deficiency was subjected to the STIA model. Using primers that hybridize within the floxed Exon 2 of *Wnt5a* (Additional file 1: Figure S2A), the Wnt5a cKO mice exhibited >90% decrease in *Wnt5a* in the paws of Wnt5a cKO mice after 2 weeks of tamoxifen-induced ablation (Additional file 1: Figure S2A) compared with littermate controls. Baseline measures in healthy Wnt5a cKO mice did not demonstrate alterations in inflammatory markers (Additional file 1: Figure S2B), in ankle morphology (Additional file 1: Figure S3), or in osteoclast activation (Additional file 1: Figure S4). Following STIA induction of RA, there was attenuated development of arthritis in the Wnt5a cKO mice in terms of ankle thickness, which plateaued below the thickness developed in the littermate controls and resolved in a similar timeframe (Fig. 1a). In a separate cohort of animals, the arthritic process was examined in control and Wnt5a cKO mice at the earliest peak of inflammation (d7). Measurement of the disease severity was performed by a pathologist blinded to genotype using previously established histopathologic scoring metrics [23, 24], using H&E stained sections from control and Wnt5a cKO mice at d7 (Fig. 2a). The Wnt5a cKO mice displayed statistically significant reduction in overall disease severity (overall H&E score) and this cohort exhibited a significant reduction in clinical score (Fig. 2b). Histologic analysis of the arthritic joints also revealed that Wnt5a cKO mice exhibit significant reductions in some parameters of inflammation, including measures of reduced extra-articular inflammation and PMN infiltration (Fig. 2b). Although not statistically different, trends of reduced pannus formation, synovial lining average and bone erosion were also observed in the Wnt5a cKO mice compared to control mice (Fig. 2b and Additional file 1: Figure S5). Numerous studies have reported that Wnt5a promotes cytokine expression [13–16]. However, only trends of decreased *TNFα*, *IL6*, and *IL1β* were observed in Wnt5a cKO at day 7 following RA induction (Additional file 1: Figure S6A), which were not statistically significant.

### Decreased osteoclast activity in arthritic Wnt5a cKO mice

**Fig. 3:**
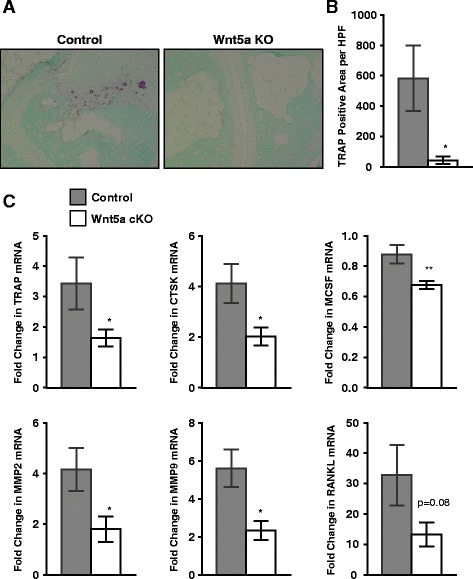
Whole body inducible Wnt5a knockout (*Wnt5a cKO*) mice have reduced osteoclast activity after induction of arthritis. **a** Tartrate-resistant acid phosphatase (*TRAP*) staining, indicated by *purple color* in control (*left panel*) and Wnt5a cKO (*right panel*), indicates fewer areas of osteoclast activity in the Wnt5a cKO mice at day 7. **b** Quantification of the percent of TRAP-positive area revealed reduced osteoclast activity in the Wnt5a cKO (*white bars*) compared to the controls (*gray bars*). **c** The levels of mRNA for the *TRAP*, *Cathepsin K* (*CTSK*), *macrophage colony-stimulating factor* (*MCSF*), *matrix metalloproteinase 2* (*MMP2*) and *MMP9* were reduced in the paws from the arthritic Wnt5a cKO mice compared to the arthritic controls. *P ≤ 0.05. A trend toward reduced *RANKL* transcript was noted

We next evaluated the osteoclast activity in the arthritic ankles from control and Wnt5a cKO mice. TRAP staining (Fig. 3a) demonstrated robust activity in the control mice, with obvious staining at multiple destruction pits, whereas Wnt5a cKO mice displayed a markedly reduced TRAP-positive area as quantified in Fig. 3b. Consistent with these findings, decreased levels of TRAP and *cathepsin K* (*CTSK*) mRNA were detected in the arthritic paws of Wnt5a cKO mice (Fig. 3c), but there were no changes in the *calcitonin receptor* (*CAL*) or *carbonic anhydrase 2 *(*CAR2*) mRNA in Wnt5a cKO mice (Additional file 1: Figure S6B). There was a trend toward lower expression of the osteoclast differentiation signal *RANKL* and reduction in the expression of *MCSF* observed in the arthritic paws of Wnt5a cKO mice (Fig. 3c). Consistent with their roles both in inflammation and in osteoclast activity, levels of *MMP2* and *MMP9* mRNA were significantly decreased in the arthritic paws of Wnt5a cKO mice (Fig. 3c).

### Wnt5a promotes osteoclast fusion in vitro

**Fig. 4:**
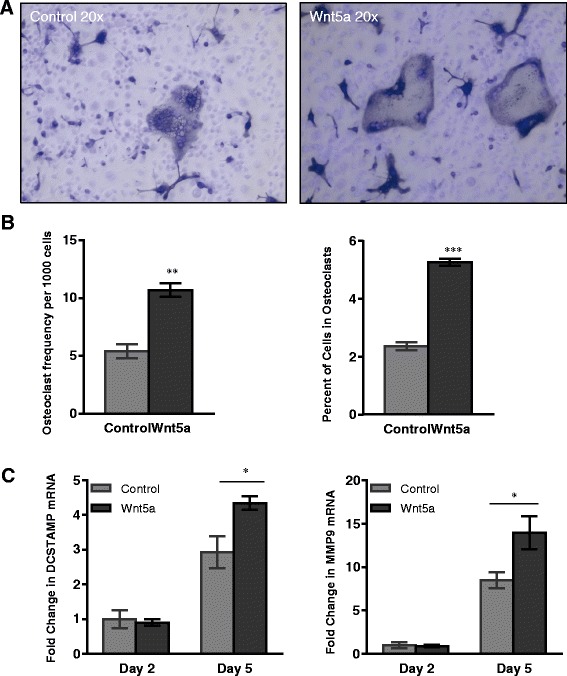
Wnt5a promotes osteoclast formation through enhanced *dendrocyte-expressed seven transmembrane protein* (*DCSTAMP*) and *matrix metalloproteinase 9* (*MMP9*) expression. Osteoclasts were formed *in vitro* from bone marrow-derived monocytes in L cell conditioned medium (*Control*) or L Wnt5a conditioned medium (*Wnt5a*). Osteoclasts were visualized after 5 days of RANKL stimulation by tartrate-resistant acid phosphatase (TRAP) (*dark color*) and 4',6-diamidino-2-phenylindole (**a**). Osteoclasts were enumerated by identifying TRAP+ cells with three or more nuclei and expressed as frequency per 1000 cells or by percent of nuclei in osteoclasts (**b**). By both measures, Wnt5a increased the osteoclast formation (**p ≤ 0.01, ***p ≤ 0.001). **c** mRNA isolated from fusing osteoclast cultures at day 2 and day 5 were analyzed for *DCSTAMP* and *MMP9* expression. The levels were increased by Wnt5a treatment. *P ≤ 0.05 for comparison of control and Wnt5a-treated cultures

To attain a deeper understanding of the mechanism of Wnt5a action in the process of osteoclastogenesis, in vitro analyses were performed to determine whether Wnt5a has an intrinsic ability to promote osteoclast fusion. MCSF-dependent BMDM were stimulated to fuse in the presence of L cell medium conditioned by cells that were engineered to overexpress Wnt5a (Wnt5a) or control media (conditioned by L cells without Wnt5a overexpression). TRAP-positive (dark color) multinucleated osteoclasts formed after 5 days of RANKL stimulation (Fig. 4a). Osteoclast fusion was stimulated by Wnt5a, both in terms of total incidence (osteoclast frequency per 1000 cells) and in the percent of nuclei fused into osteoclasts (Fig. 4b). In contrast, Wnt5a did not significantly change the nuclei per osteoclast total or total nuclei in osteoclasts (Additional file 1: Figure S7).

Transcripts were analyzed in fusing BMDM cultures after 2 and 5 days of RANKL stimulation to further evaluate the impact of Wnt5a on osteoclast fusion. *Wnt5a* mRNA levels were not altered during the fusion process (Additional file 1: Figure S8). Treatment with Wnt5a did not significantly alter the expression of osteoclast genes (*CTSK* or *TRAP*) or an inhibitor of *osteoclastogenesis* (*OPG*) (Additional file 1: Figure S8). However, Wnt5a promoted the expression of *DCSTAMP* (Fig. 4c), a cell surface molecule that is essential for osteoclast fusion [30]. Consistent with observations in the STIA model, fusing macrophages treated with Wnt5a expressed increased levels of *MMP9* but not *MMP2* (Fig. 4d). In contrast, Wnt5a did not affect the expression of other nuclear factor activating T cells 1c (NFATc1) downstream genes, including *MMP14*, *CLCN7*, *CAR2*, *RCAN2* and *OSCAR*, or *NFATc1* itself (Additional file 1: Figure S9).

## Discussion

Wnt5a is elevated in the synovium of RA patients [18], and enhanced Wnt5a secretion by fibroblast-like synoviocytes has been attributed to their persistent activation [19]. Thus, we sought to provide genetic evidence for a causal link between Wnt5a and RA development. The present study utilized a mouse model in which ablation of Wnt5a is inducible, thereby avoiding the embryonic lethality of the conventional knockout strain [21]. This inducible ablation model also permits the ablation of Wnt5a proximal to the induction of arthritis, thereby eliminating the confounding effects of Wnt5a in bone development [20] (Additional file 1: Figure S1-S3). Collectively, this study shows that mice deficient for Wnt5a are resistant to development of RA-like disease in the STIA model.

The acute genetic ablation of Wnt5a in the STIA model led to reductions in some markers of inflammation. Based upon histologic analysis, Wnt5a cKO mice exhibited statistically significant reductions in overall inflammatory status, extra-articular inflammation and PMN cell infiltration (Fig. 2). In this regard, it is well-appreciated that neutrophils are important mediators in the development of arthritis [31, 32]. Failure to recruit neutrophils correlates with reduction in the overall progression of disease severity in other models of arthritis [33]. Further, since Wnt5a is described to be chemotactic to neutrophils [34], it is possible that this is a significant component of the mechanism by which Wnt5a cKO mice are protected from the development of STIA.

Using the Wnt5a cKO mouse model, we recently reported that Wnt5a ablation protects against adipose tissue inflammation and systemic metabolic dysfunction that is associated with diminished levels of monocyte chemoattractant protein 1 (MCP-1), IL6 and TNFα in the adipose tissue [10]. Although these cytokines are shared in the pathogenesis of metabolic dysfunction and RA [1, 5], only trends towards reduction of *TNFα*, *IL6*, and *IL1β* transcripts were observed in the arthritic paws from the Wnt5a cKO mice (Additional file 1: Figure S6) as was a trend toward diminished lymphocyte infiltration in the histological sections (Additional file 1: Figure S5). In total, these data are indicative that Wnt5a is one of many modulators of inflammation in the RA model, which can be compensated for by other inflammatory molecules. However, there appears to be a modest and appreciable protection from the inflammation in RA conferred by Wnt5a deficiency. Further, we acknowledge the possibility that Wnt5a has a more profound impact on inflammation in the STIA model at other time points.

In contrast to the increase in Wnt5a expression in the synovium of RA patients [18], we found a reduction in the amount of *Wnt5a* in the joints from mice undergoing STIA. Beyond species differences, there are a number of possible explanations for this discrepancy. First, there was a concurrent increase in the Wnt5a co-receptor *ROR2* (Additional file 1: Figure S1), indicating that reduced amounts of Wnt5a might be sufficient to drive RA pathology. In this regard, Wnt5a is highly glycosylated and is believed to exhibit its effects through short-range paracrine interactions [35, 36]. Thus, localized changes in Wnt5a may be sufficient to modulate RA development, particularly when paired with a concomitant increase in *ROR2*. It should be also noted that our analysis was performed using the entire joint; including fibroblast-like synoviocytes, bone, bone marrow, and extra-articular inflammatory tissue. Indeed, although Wnt5a is reported to be among the most highly expressed Wnt homologs expressed in bone, immunohistochemical analysis demonstrated that it is predominantly expressed by osteoblast/osteoclasts and not the osteocyte [20]. Thus, further exploration of Wnt5a in the arthritic space, beyond the synovial tissue, is warranted.

Wnt5a has been functionally implicated in the development of RA by a study that delivered a soluble version of ROR2, a putative Wnt5a receptor, to mice in the collagen-induced arthritis (CIA) model [20]. Although mice receiving the soluble ROR2 were not protected from development of ankle thickness or clinical score, they did exhibit reduced radiographic damage [20], consistent with a reduction in osteoclast activity. Our work supports and extends these observations in several ways. First, using the Wnt5a cKO mouse model we provide direct mouse genetic evidence to support that Wnt5a plays a role in the pathogenesis of RA. Second, the inducible Wnt5a cKO mice were used to ablate Wnt5a immediately prior to the induction of the STIA model, which eliminates the confounding effects of Wnt5a-deficiency on long-term bone development [20, 37–39]. Third, we document an effect of Wnt5a in the STIA model, which recapitulates the initiating events of the disease with a higher penetrance than the CIA model. Notably, the STIA model also avoids the use of adjuvant that can potentially act as a confounder. Finally, our study provides mechanistic detail about the role of Wnt5a in osteoclast fusion.

Macrophage cell fusion is required for the formation of multinucleated osteoclasts that function in the catabolism of bone matrix. Osteoclast formation from myeloid precursors is regulated by a sequence of molecular events involving the cell surface receptors including DCSTAMP, CD36, CD47, TREM2 and E-cadherin [40]. Additionally, osteoclast-specific genes including MMP9, CTSK and TRAP [41], are also required for cell fusion during osteoclastogenesis. Although it has been implicated in differentiation of multiple cell types, Wnt5a is not widely appreciated to promote cellular fusion. In the present study, we find that exogenous Wnt5a added to fusing BMDM enhanced their fusion into osteoclasts and upregulated *DCSTAMP* (Fig. 4), a cell surface molecule essential for formation of osteoclasts in vivo [30]. *MMP9* was also found to be upregulated by Wnt5a in fusing osteoclasts (Fig. 4c). MMP9 has been shown to be critical for the development of arthritis as MMP9 KO mice develop less inflammation and joint destruction in the serum transfer model [42]. MMP9 has also been shown to modulate several aspects of osteoclast function, including enhancement of cell migration, potentiation of cell fusion, and increased bone metabolism [43–46]. Altogether, our data indicate that Wnt5a promotes osteoclast formation, and suggests a possible role of Wnt5a in development of other bone resorption disorders.

Rheumatoid arthritis is associated with cardiometabolic diseases [3, 4], and a growing number of animal studies have investigated this linkage. Assessment of arthritis development in ApoE-deficient mice using the CIA model has led to conflicting reports on the consequences of hypercholesterolemia on promoting arthritis [47, 48]. However, using a modified chronic STIA model, more recent evidence demonstrated that ApoE-deficient mice display aggravated development of arthritis but not an increased plaque burden [49]. Conversely, when placed on an atherogenic diet, K/BxA^g7^ mice display increased plaque burden [50]. The present study suggests that Wnt5a might be a possible common mechanism linking both RA and cardiovascular disease. It has been shown that Wnt5a contributes to increased cardiac dysfunction following ischemia/reperfusion injury [11] and to impaired revascularization in a model of peripheral arterial disease [9]. Moreover, previous research demonstrated that Wnt5a impairs metabolic function at least in part through its role in exacerbating adipose tissue inflammation in the obese state [10, 12]. Wnt5a is markedly upregulated in visceral adipose tissue (VAT) and correlates strongly with indices of inflammation and diabetes mellitus [10, 51]. Despite the existence of “rheumatoid cachexia” in late-stage disease, large scale meta-analysis reveals that obesity is a risk factor for RA that is associated with disease severity [52, 53]. It has been proposed that adipokines, cytokines secreted by adipose tissue, are molecular drivers of both diseases [52, 54]. RA patients exhibit elevated adipokine levels, including leptin, adiponectin, and resistin, in serum or synovial fluid compared to healthy patients [52, 55–57], and these factors frequently correlate with disease progression [58, 59]. Collectively, these observations warrant further study to establish whether Wnt5a functions as a putative nexus target in RA and cardiometabolic diseases.

Recent advances in DMARDs have revolutionized the treatment landscape for RA patients. However, cessation of treatment inevitably leads to relapse. Furthermore, a subset of patients remains refractory to these therapeutics agents. Current treatment modalities focus on inhibition of the inflammatory process and do not directly target osteoclast activity. Based upon the multi-modal influence of Wnt5a on RA etiology, specifically as a modulator of osteoclast activity and inflammation, it may represent a novel target that has potential to modulate both arms of RA disease progression.

## Conclusions

Using a genetic model, this study demonstrates for the first time that loss of Wnt5a attenuates the development of RA. Wnt5a functions in two major aspects of RA pathology; through enhancement of some parameters of inflammation and by promoting osteoclast fusion. These results are the first to demonstrate a role of Wnt5a in autoimmune disease progression or in cell-cell fusion.

## Additional file

Additional file 1: Figure S1. Gene expression of Wnt5a and ROR2 during the development of STIA. **Figure S2.** Characteristics of the baseline phenotype of control and Wnt5a cKO mice. **Figure S3.** Histology of the baseline phenotype of control and Wnt5a cKO mice. **Figure S4.** Baseline osteoclast phenotype of control and Wnt5a cKO mice. **Figure S5.** Additional histopathological scoring of arthritis in Wnt5a cKO mice. **Figure S6.** Gene expression of cytokines and osteoclast genes in paws of control and Wnt5a cKO mice undergo﻿ing STIA. **Figure S7.** Characteristics of fusing osteoclasts in the absence and presence of Wnt5a-treated medium. **Figure S8.** Gene expression of osteoclast markers during BMDM differentiation to osteoclasts. **Figure S9.** Gene expression of NFATa1 markers during BMDM differentiation to osteoclasts. (PDF 4647 kb)

## Abbreviations

ANOVA: Analysis of variance
BMDM: Bone marrow-derived macrophages
CAL: Calcitonin receptor
CAR2: Carbonic anhydrase 2
CIA: Collagen-induced arthritis
CLCN7: Chloride voltage-gated channel 7
CTSK: Cathepsin K
DAPI: 4',6-Diamidino-2-phenylindole
DCSTAMP: Dendrocyte-expressed seven transmembrane protein
DMARD: Disease-modifying anti-rheumatic drug
DMEM: Dulbecco’s modified Eagle’s medium
FBS: Fetal bovine serum
H&E: Hematoxylin and eosin
HPF: High-powered field
HPRT: Hypoxanthine guanine phosphoribosyl transferase
IACUC: Institutional Animal Care and Use Committee
KO: Knockout
MCP1: Monocyte chemoattractant protein 1
MCSF: Macrophage colony-stimulating factor
MMP2: Matrix metalloproteinase 2
MMP9: Matrix metalloproteinase 9
MMP14: Matrix metalloproteinase 14
NFAT1c: Nuclear factor activating T cells 1c
OPG: Osteoprotegerin
OSCAR: Osteoclast-associated immunoglobulin-like receptor
PMN: Polymononuclear
RA: Rheumatoid arthritis
RANKL: Signal receptor activator of nuclear factor-kB ligand
RCAN2: Regulator of calcineurin 2
ROR2: Receptor-tyrosine orphan receptor 2
STIA: Serum transfer-induced arthritis
TNFα: Tumor necrosis factor alpha
TRAP: Tartrate resistant acid phosphatase
Wnt5a: Wingless-type MMTV integration site family, member 5a
