# Supplementary material for: Genetic deficiency of Wnt5a diminishes disease severity in a murine model of rheumatoid arthritis
Source: Arthritis Res Ther. 2017 Jul 19;19:166. doi: 10.1186/s13075-017-1375-0 (PMC5518154; doi:10.1186/s13075-017-1375-0)

Figure S1: Normalized mRNA expression from paw joints isolated from healthy Control mice prior to STIA induction (Baseline) and littermate controls 7 days following STIA induction (RA d7) for Wnt5a and ROR2. **** indicates p≤0.0001 and ** indicates p≤0.01

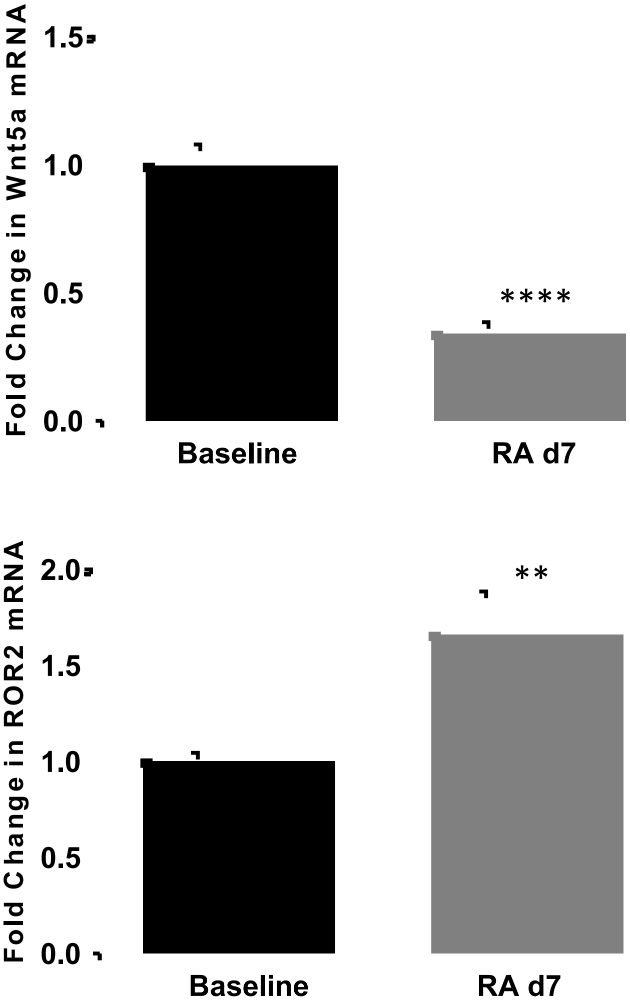

Figure S2: *Characterization of baseline Wnt5a cKO mice. (*A) Allele structure of the Wnt5a cKO mice indicates the position of the flox sites around Wnt5a Ex2. Wnt5a cKO was verified by mRNA analysis of the paw joints, and the levels of Wnt5a were reliably reduced by at least 90% in all cre + animals. **** indicates p≤0.0001. Primers specific to Exon 2 were used to assess deletion (relative position of binding sites indicated by blue lines in the flox diagram). (B) mRNA from d7 paws from healthy Control and Wnt5a cKO mice was analyzed for MMP2, MMP9, IL1β, IL6, MCP1 and TNFα. There was no significant difference between the levels in Control and Wnt5a cKO mice prior to induction of STIA at baseline (no STIA).

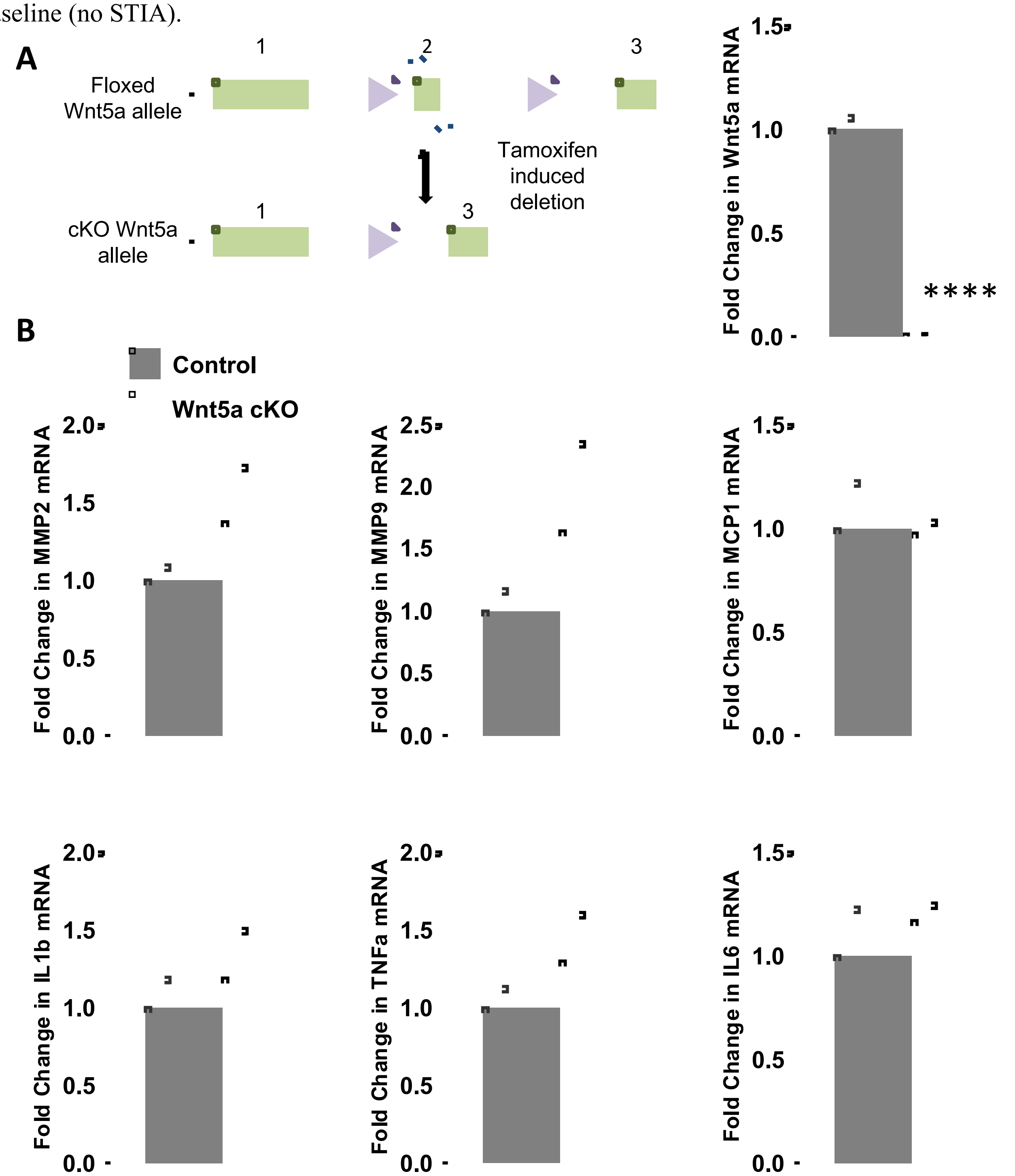

Figure S3: H&E staining of sections from Control (left panels) and Wnt5a cKO (right panels) in healthy ankles, prior to arthritis induction demonstrates no overt phenotype in the Wnt5a cKO at baseline.

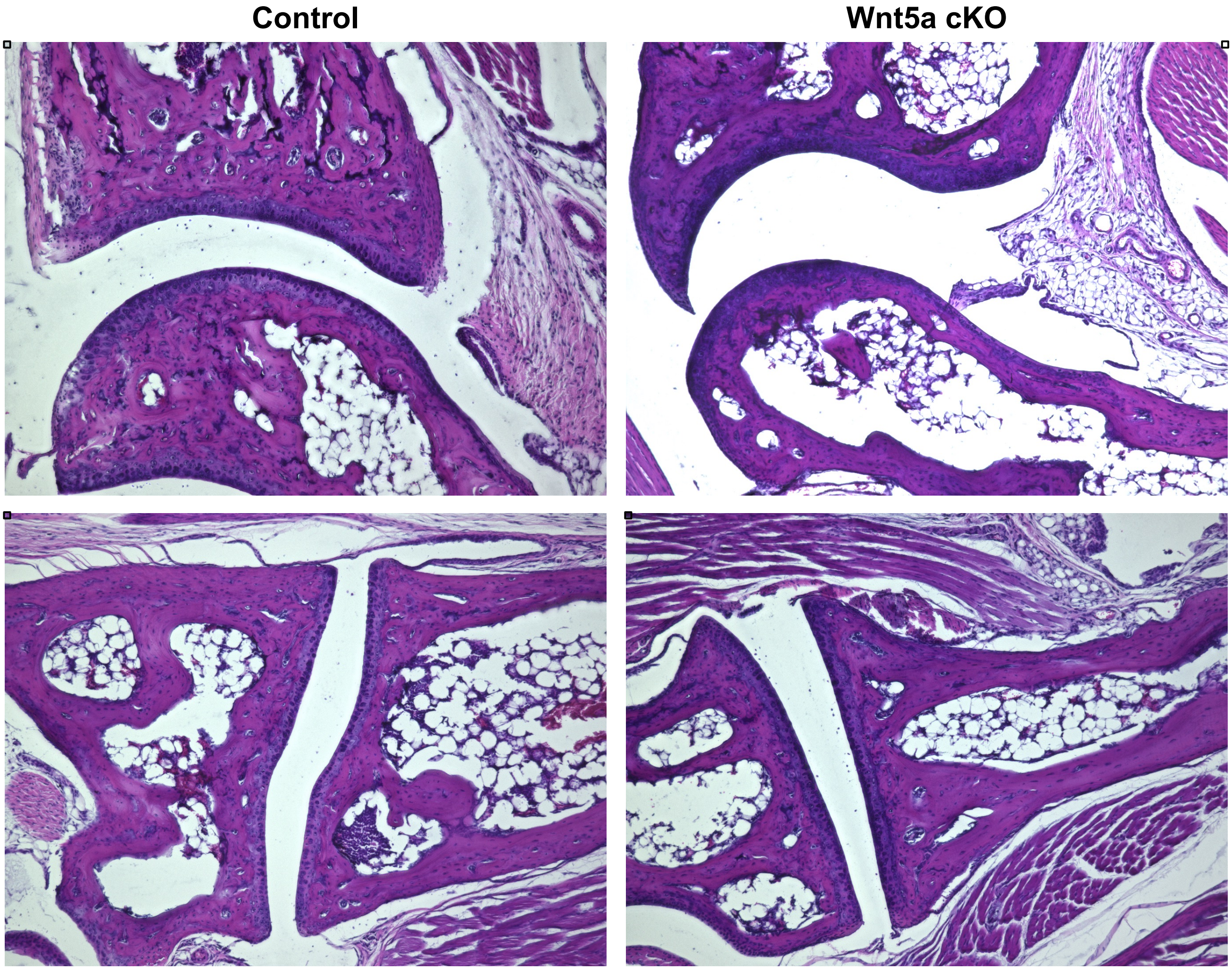

Figure S4: *No baseline osteoclast phenotype in the Wnt5a cKO mice.* (A) TRAP stained sections from ankles of Control and Wnt5a cKO mice show a lack of robust osteoclast activity in healthy bones. (B) mRNA from paws from Control and Wnt5a cKO mice which did not receive K/BxN serum were analyzed for markers of osteoclast differentiation and activity. There was no significant difference between the levels in Control and Wnt5a cKO mice.

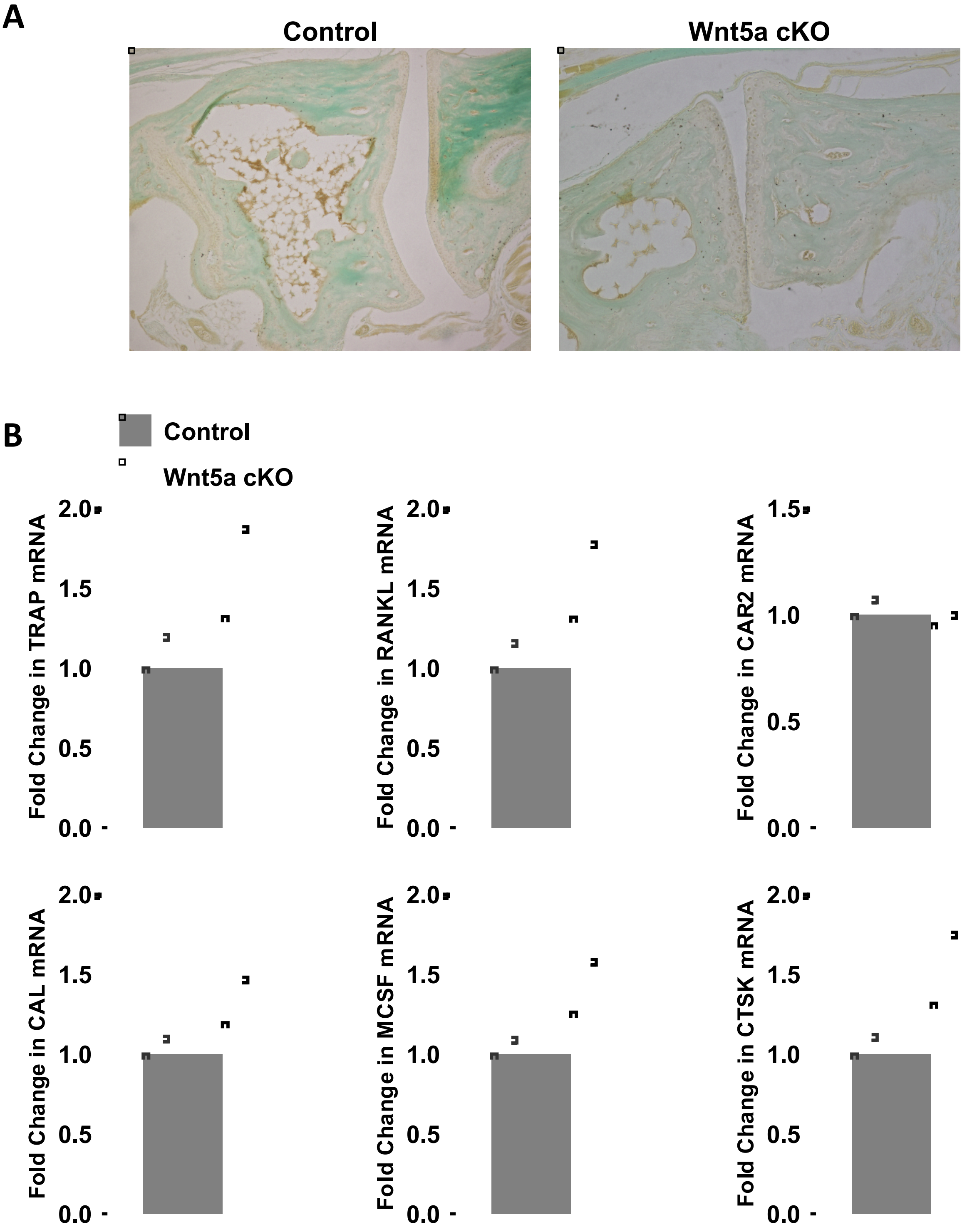

Figure S5: Histopathological score of Pannus formation and Synovial Lining in the STIA model.

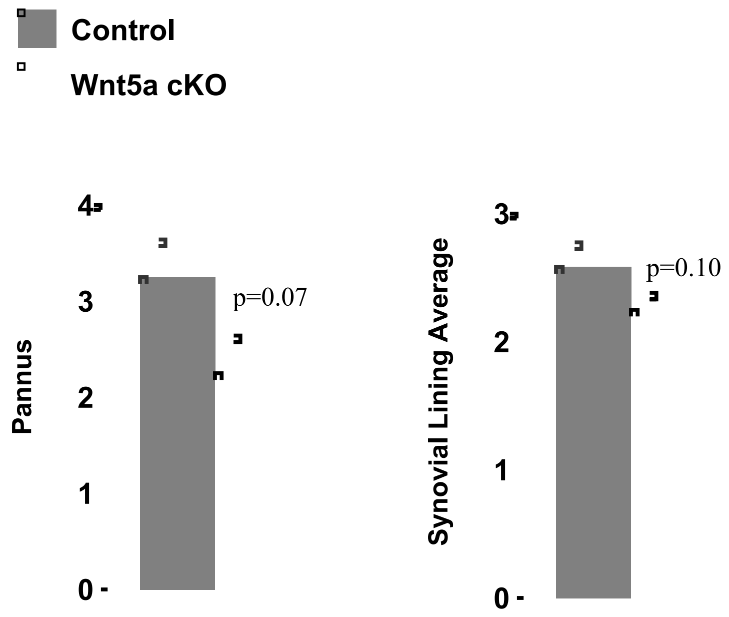

Figure S6 *Levels of arthritis modulators in control and Wnt5a cKO mice in the STIA model*
mRNA from d7 paws from Control and Wnt5a cKO mice was analyzed for (A) IL1β, IL6, MCP1 and TNFα and (B) CAL and CAR2. There was no significant difference between the levels in Control and Wnt5a cKO mice.

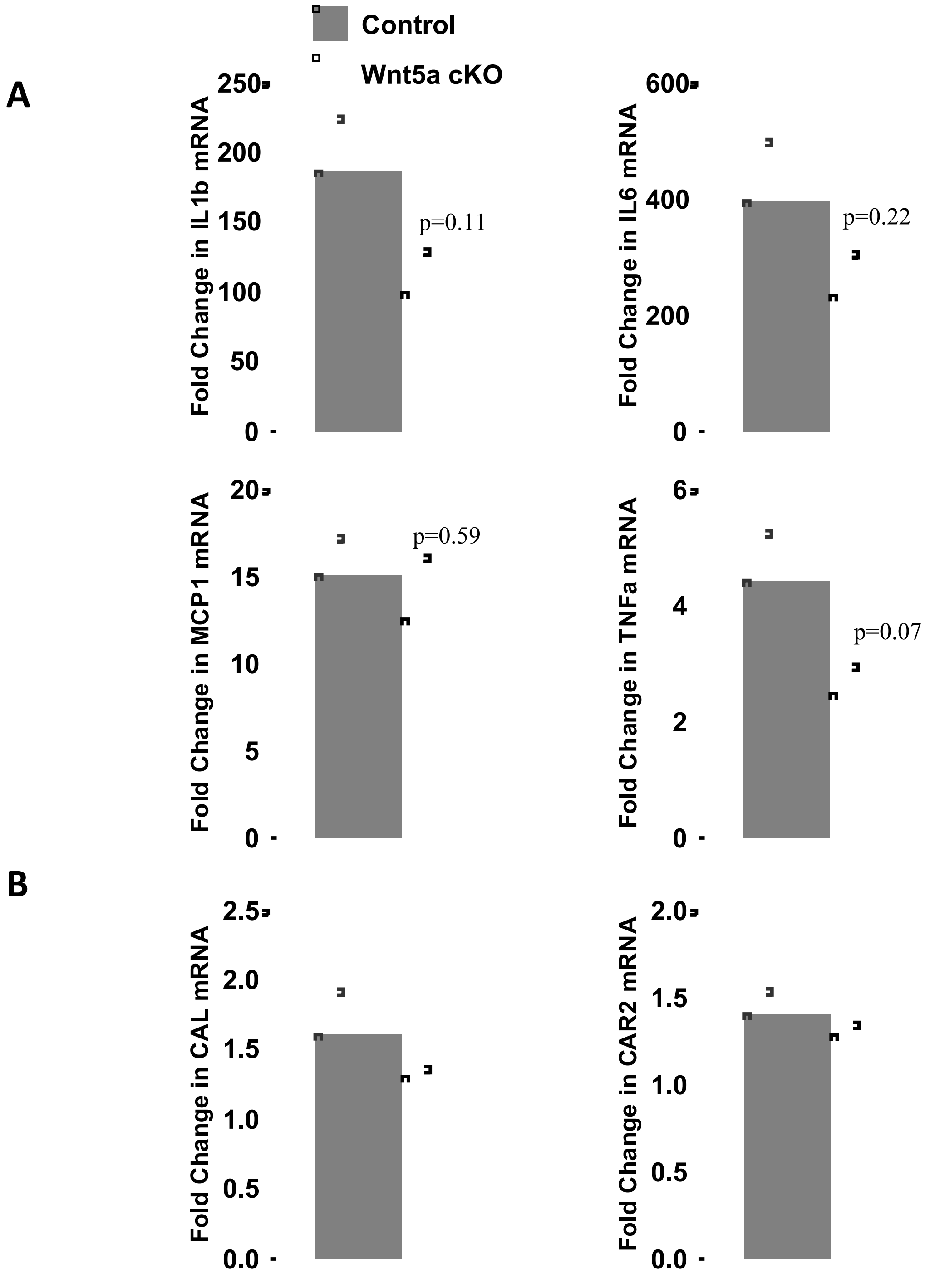

Figure S7: Characteristics of the fusing osteoclasts in the absence and presence of Wnt5a.

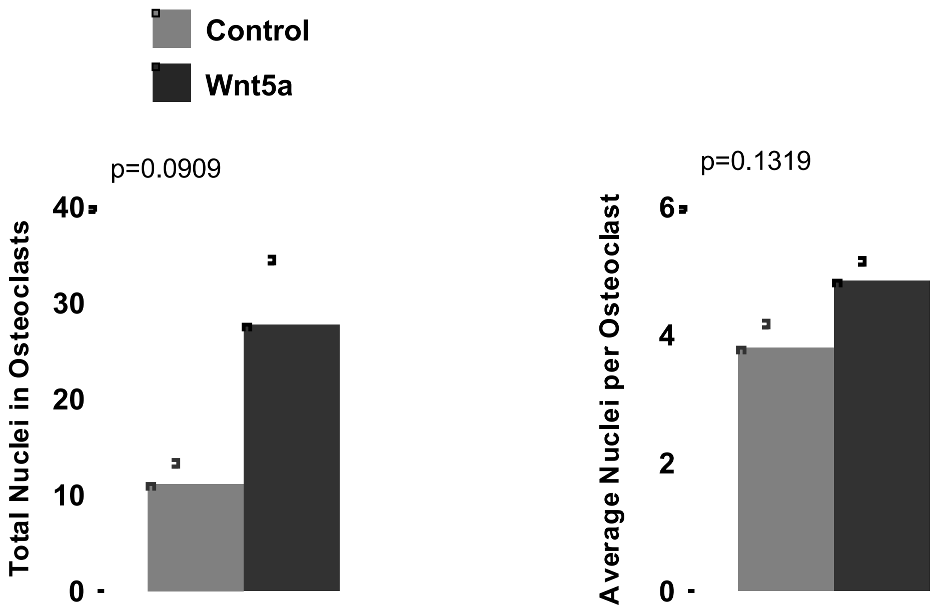

Figure S8: Osteoclast marker expression during fusion of BMDM to osteoclasts in the presence or absence of Wnt5a.

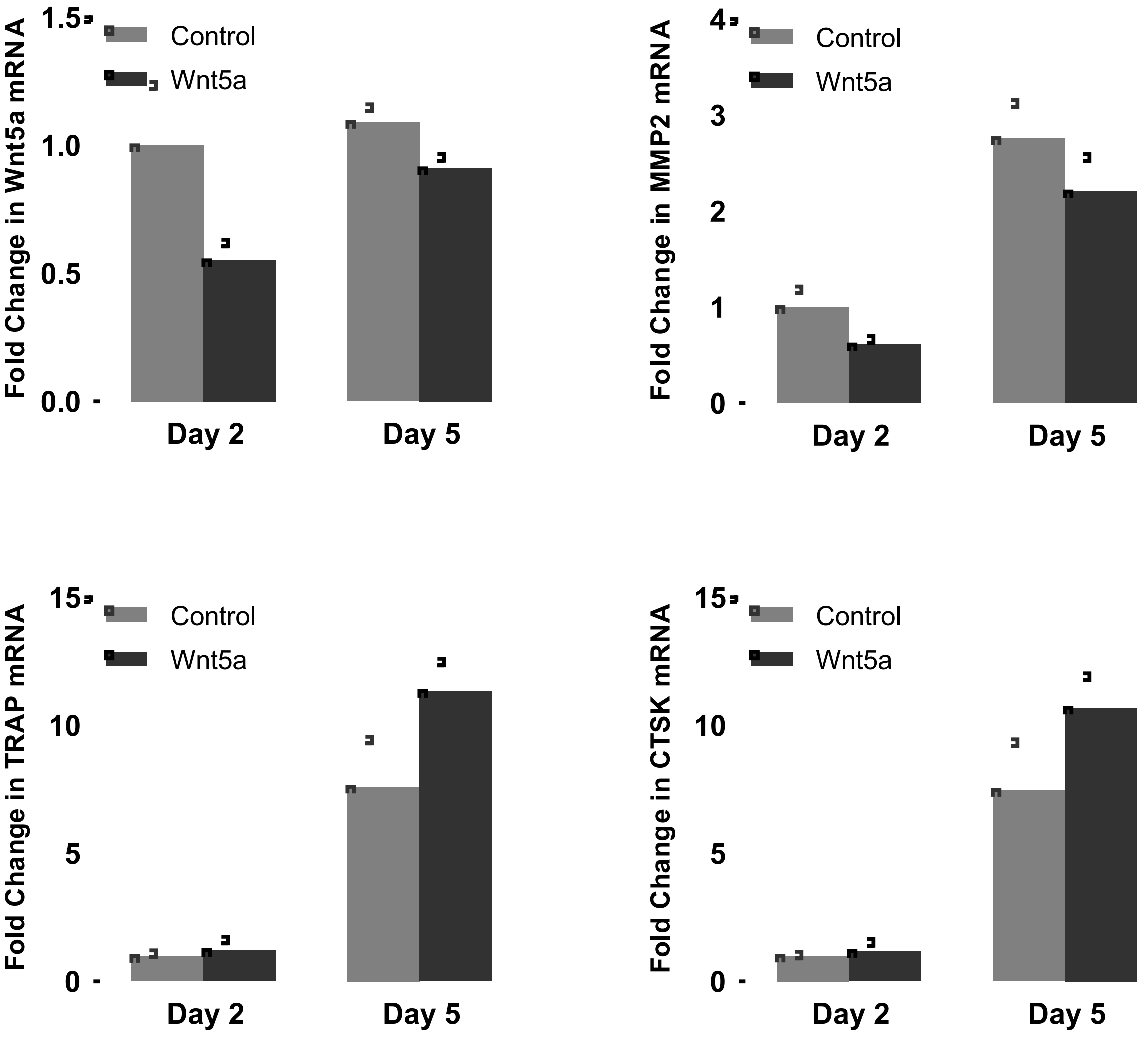

Figure S9: Marker expression levels during fusion of BMDM in the presence or absence of Wnt5a.

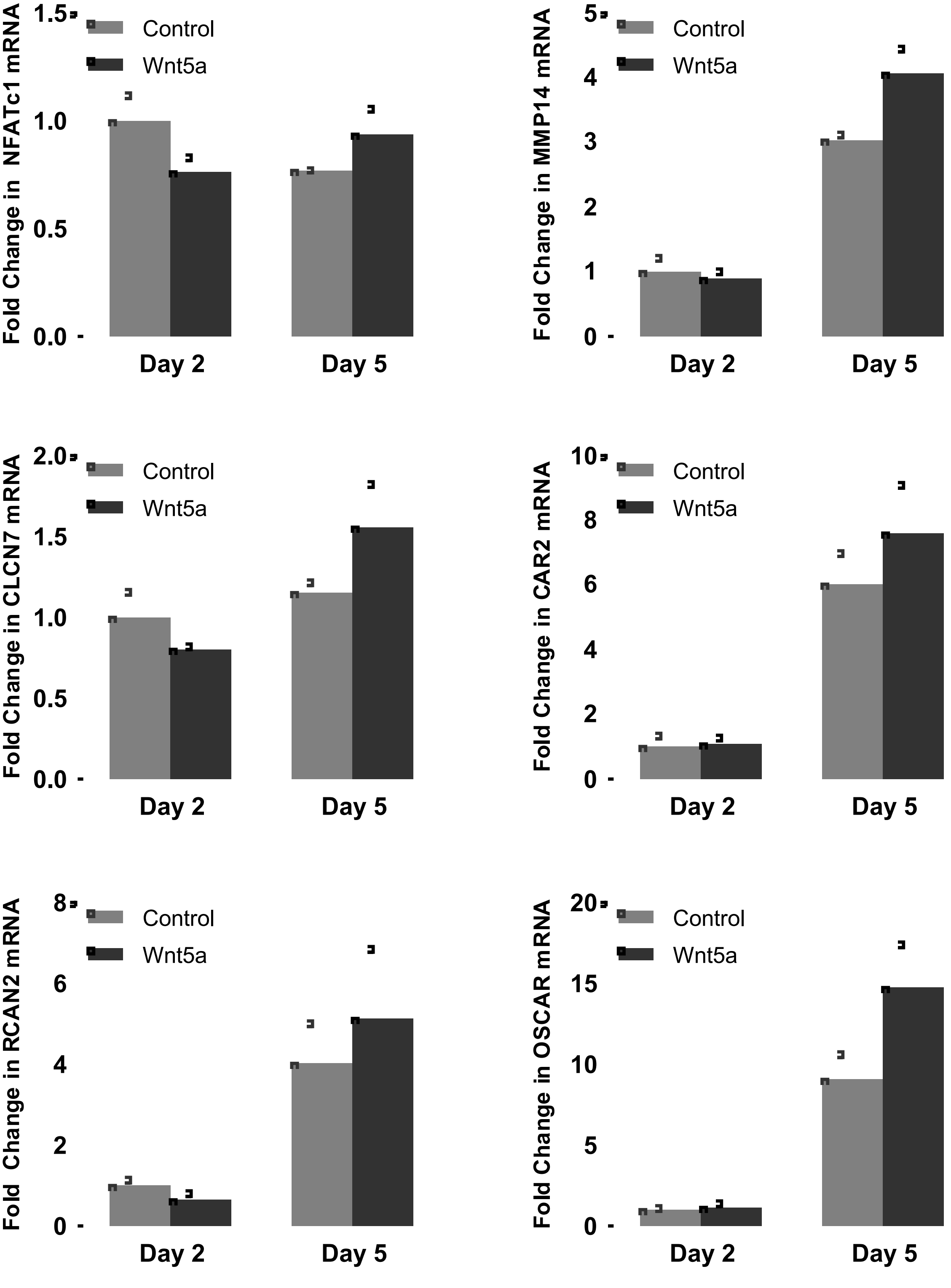

Supplement: Additional file 1: Figure S1. — Gene expression of Wnt5a and ROR2 during the development of STIA. Figure S2. Characteristics of the baseline phenotype of control and Wnt5a cKO mice. Figure S3. Histology of the baseline phenotype of control and Wnt5a cKO mice. Figure S4. Baseline osteoclast phenotype of control and Wnt5a cKO mice. Figure S5. Additional histopathological scoring of arthritis in Wnt5a cKO mice. Figure S6. Gene expression of cytokines and osteoclast genes in paws of control and Wnt5a cKO mice undergo﻿ing STIA. Figure S7. Characteristics of fusing osteoclasts in the absence and presence of Wnt5a-treated medium. Figure S8. Gene expression of osteoclast markers during BMDM differentiation to osteoclasts. Figure S9. Gene expression of NFATa1 markers during BMDM differentiation to osteoclasts. (PDF 4647 kb) [file 13075_2017_1375_MOESM1_ESM.pdf]
